# Evaluation of GeneXpert MTB/Rif Ultra assay performance on formalin-fixed paraffin-embedded tissues for Mycobacterium tuberculosis detection

**DOI:** 10.1099/jmm.0.001918

**Published:** 2024-10-22

**Authors:** Calvin Ka-Fung Lo, Dale Purych, Inna Sekirov, Jaswinder Khattra, Trevor J. Hird, Shazia Masud

**Affiliations:** 1 Department of Pathology and Laboratory Medicine, University of British Columbia, Vancouver, British Columbia, Canada; 2 Department of Pathology and Laboratory Medicine, Surrey Memorial Hospital, Fraser Health, Surrey, British Columbia, Canada; 3 BC Centre for Disease Control, Public Health Laboratory, Vancouver, British Columbia, Canada

**Keywords:** extra-pulmonary, formalin-fixed tissue, MTB, *Mycobacterium tuberculosis*, resistance screening, Xpert Ultra

## Abstract

We evaluated the Xpert MTB/Rif Ultra assay performance for *Mycobacterium tuberculosis* (MTB) detection in formalin-fixed paraffin-embedded tissue (FFPET) compared to mycobacterial culture or laboratory-developed MTB PCR test (LDT). FFPET samples with histological features suggestive of tuberculosis from 2018 to 2023 were selected. Five hundred microlitres of tissue lysis buffer was added to FFPET scrolls and incubated at 75 °C for 5 min. After adding 50 µl of proteinase K and overnight incubation at 56 °C, sample aliquots were processed as per the manufacturer’s instructions. MTB culture or LDT assay results were used as a reference for sensitivity and specificity calculations. Of 51 eligible FFPET, 32 were positive for MTB either by culture or LDT PCR on FFPET. Xpert MTB/Rif Ultra detected MTB in 23/32 positive specimens [71.9%, 95% confidence interval (CI) 54.6–84.4%]. Of nine discordant specimens, seven were MTB positive by culture and two were identified by LDT MTB PCR only, as no specimen was submitted for MTB culture. Of 19 negative samples, 100% specificity (95% CI 83.2–100.0%) was attained via Xpert MTB/Rif Ultra. Implementation of Xpert MTB/Rif Ultra on FFPET within clinical laboratories is promising, given its improved turnaround time compared to MTB culture and ability to detect MTB in cases where no tissue is available for culture.

**Impact Statement**

Extra-pulmonary tuberculosis remains a challenging diagnosis, given the paucibacillary nature of the disease and situations where no fresh tissue samples were collected during a surgical procedure or biopsy for mycobacterial culture. Furthermore, current commercial molecular testing has not fully evaluated the performance against preserved tissue samples and is approved for testing of respiratory specimens (e.g. sputum). Hence, we conducted a study with our provincial reference laboratory using a molecular testing platform (Xpert MTB/Rif Ultra). This platform has specific targets for *Mycobacterium tuberculosis* (MTB, the bacteria causing tuberculosis disease) and can effectively detect the presence of its DNA within 2 h after overnight sample preparation. To our knowledge, we are the first Canadian site conducting a formal pilot evaluation of this molecular testing platform against this specimen type (formalin-fixed, paraffin-embedded tissues). Should it be comparable to the reference standard of MTB culture and reference laboratory in-house molecular testing, this study will help support the implementation of this rapid molecular testing into routine clinical laboratories for MTB diagnosis when there is no specimen for MTB culture.

## Data availability

Data and materials are available and will be provided upon request from the corresponding author.

## Introduction

Extra-pulmonary tuberculosis (EPTB) globally represents about 17% of new and relapsed cases of tuberculosis (TB), according to the WHO 2023 Global Tuberculosis Report [1]. Diagnosis of EPTB remains challenging for various reasons. The current reference standard for *Mycobacterium tuberculosis* (MTB) diagnosis remains culture, as it enables gold-standard phenotypic drug susceptibility testing [2] and maximizes sensitivity for this organism. The MTB culture requires fresh tissue sample, long turnaround times of up to 6–8 weeks and biosafety considerations, including containment level 3 (CL3) laboratory settings for propagative activities (e.g. antimicrobial susceptibility testing) [3,4]. The gold standard of practice is to split biopsied samples into a fresh specimen for microbiological culture and a formalin-fixed specimen for histopathological examination. However, in cases when MTB (or any other infectious process) is not initially high on the differential diagnosis, biopsied specimens are sent in their entirety to Anatomical Pathology within formalin (with no part of the specimen allocated for MTB culture). Histopathological features of TB infection include necrotizing granulomatous inflammation and presence of acid-fast bacilli (AFB) [5]. However, TB can also present with other inflammatory patterns, such as non-necrotizing granulomatous processes and suppurative inflammation [6]. Furthermore, multiple mimics exist, including non-tuberculous mycobacteria (NTM), bacterial or fungal infections and non-infectious conditions (e.g. sarcoidosis and vasculitides) [6]. In cases where the whole biopsy is formalin-fixed, no culture is set up typically; however, if MTB is suspected post-factum, formalin-fixed paraffin-embedded tissue (FFPET) may be the only specimen available for microbiological testing. Should histologic findings be equivocal or non-specific for MTB disease, a definitive diagnosis might not be provided. This leads to diagnostic delays, need for repeat invasive sampling procedures, increased healthcare costs and delays in both specialist consultation and initiation of care.

Xpert MTB/Rif assay (Cepheid, USA) is a semi-nested real-time PCR-based assay targeting *rpoB* (Rv0664), which is FDA (Food and Drug Administration) approved for detecting MTB in sputum specimens. In a single cartridge, specimens undergo sample lysis, DNA extraction and amplification followed by detection of MTB and rifampin resistance without the need for level 3 containment laboratory. Based on a systematic review of 36 studies evaluating the performance of this assay for lymph nodes, Xpert MTB/Rif had pooled sensitivity (SNS) of 78% [compared to composite reference standard (CRS)] and 84% (compared to culture) [7]. Pooled specificity (SPC) was 98% against CRS and 91% against culture, although significant heterogeneity existed across CRS [7]. A 2018 Cochrane review summarized the accuracy of Xpert MTB/Rif assay in EPTB specimens: across 66 studies evaluating 16 213 specimens, pooled sensitivity (defined by culture) was quite variable depending on specimen type (e.g. 31% in pleural tissue versus >80% in urine to 97% in bone or joint fluid) [8]. Meanwhile, pooled Xpert MTB/Rif assay specificity (defined by culture) had less variability (82% in bone or joint tissue up to 99% in pleural fluid, urine and peritoneal fluid) [5,8].

As formalin fixation inactivates microbiologic organisms and degrades genetic material, this renders specimens unsuitable for culture and can decrease the sensitivity of molecular detection methods. Other technical challenges include deparaffinization of collected tissue that needs to be done prior to nucleic acid extraction. While previous studies have evaluated the performance of the prior Xpert MTB/Rif assay, it is less sensitive than the newer Xpert MTB/Rif Ultra assay, which contains *IS6110* and *IS1081* targets in addition to the *rpoB* gene target [9]. Therefore, the performance of Xpert Ultra MTB/Rif assay on FFPET is likely to be superior to the earlier version of Xpert assay and deserves further scrutiny [9].

We conducted a study evaluating the performance of Xpert MTB/Rif Ultra assay to detect MTB in FFPET compared to mycobacterial culture and/or reference laboratory-developed test (LDT) PCR.

## Methods

Archived FFPET samples (2018–2023) from two pathology laboratories within the health region and a provincial reference laboratory [British Columbia Centre for Disease Control (BCCDC), Vancouver, British Columbia] were included. To be included, tissue samples needed to demonstrate histological features suggestive of TB (i.e. granulomatous inflammation, with or without AFB). The distribution of specimen types is outlined in Table 1. Some of the FFPET samples had concurrent fresh tissue specimens submitted for MTB culture at the time of tissue biopsy.

**Table 1.** Distribution of sample type in correlation with positive culture and/or LDT result (*n*=32)

Sample type	No. of samples	No. with smear-positive	MTB culture positive	LDT MTB PCR positive	GeneXpert MTB/Rif Ultra assay, with sensitivity
Lymph nodes	14	1	8	5	MTB in 23 of 32 positive specimens (i.e. SNS=71.9%, 54.6–84.4%)
Lung (parenchymal or pleural tissue)	6	2	4	2	
MSK†/joint	4	2	4	0*	
Gastrointestinal:Peritoneal (*n*=1)Omentum (*n*=1)	2	0	1	1	
Gynaecological:Fallopian tube (*n*=1)Endometrial biopsy or uterine tissue (*n*=2)	3	1	3	0	
NeuroendocrineBrain biopsy (*n*=1)Thyroid (*n*=1)	2	0	1	1	
Dermatologic	1	0	0	1	

* PCR indeterminate on one sample, musculoskeletal system.

†Musculoskeletal system.

Depending on tissue size, two to four scrolls (20 µm thickness each) were used for processing. For extraction of MTB DNA from FFPET scrolls, a procedure described by Budvytiene *et al*. was used with some minor modifications [9]. In summary, 500 µl of tissue lysis buffer (ATL Buffer, Qiagen GmbH, Hilden, Germany) was added to FFPET scrolls and incubated at 75 °C for 5 min to melt the paraffin. After centrifugation for 10 s at 6000 *g*, 50 µl proteinase K (Qiagen GmbH) was added and vortexed.

The FFPET-protein K solution was incubated overnight (~16–18 h) at 56 °C. Specimens were centrifuged for 10 s (6000 *g*) followed by 5 min of room temperature incubation to solidify the paraffin. After puncturing the paraffin surface by using 1 ml pipette tip, the liquid specimen was transferred to a new tube containing 1.5 ml of Xpert MTB/Rif Ultra sample reagent. The entire mixture (~2 ml) was processed according to the manufacturer’s instructions. Reference laboratory MTB LDT PCR and MTB culture results (where fresh tissue was concurrently submitted for mycobacterial culture) were used as comparators for sensitivity and specificity calculations [with 95% confidence interval (CI) computed]. Chi-square tests of independence were also computed against Xpert MTB/Rif Ultra for both MTB LDT PCR and MTB culture, respectively.

Regarding the reference laboratory LDT PCR, it is a real-time PCR assay that targets both *mpt64* and IS*6110* targets and was introduced into routine clinical use at BCCDC in 2011 [10]. The test is run as three separate reactions, targeting the *mpt64* and IS*6110* targets of MTB and human RNaseP gene as sample quality control. Probes are labelled with TaqMan fluorescent dyes. The institutional ethical approval was not required as the study objective served as a quality improvement project to improve the current laboratory processes for MTB diagnosis via PCR on FFPET specimens.

## Results

In total, 51 FFPET clinical specimens from 2018 to 2023 were included in the study. In total, 32 specimens (62.7%) were positive for MTB either via MTB culture or reference laboratory LDT PCR; 21 (65.6%) were confirmed by MTB growth from concurrently collected fresh tissue specimen and 11 (34.4%) confirmed by MTB LDT PCR. Among the tissue specimens positive for MTB by the reference standard, lymph nodes (*n*=14, 43.8%) and lung parenchymal or pleural tissue (*n*=6, 18.8%) were the most common specimen types. The description of positive samples is summarized in Table 1. Except for six specimens that had AFBs visualized during histopathological examination, all remaining were negative for AFBs. The Xpert MTB/Rif Ultra assay successfully detected MTB in 23 out of 32 positive specimens, with an analytical sensitivity of 71.9% (95% CI 54.6–84.4%). Xpert MTB/Rif Ultra assay successfully identified MTB in 5 of 6 AFB-positive samples, 7 of 12 AFB-negative but culture-positive specimens and 11 of 14 specimens without AFB staining results available.

Among the nine discordant results, all were AFB negative. Seven were MTB culture positive and two were MTB PCR positive by a reference laboratory. Table 2 summarizes the specific specimen types and comparison between the discordant reference standard and the Xpert MTB/Rif Ultra assay result.

**Table 2.** Analysis of discordant results via GeneXpert MTB/Rif Ultra assay (*n*=9)

Discordant isolate – specimen type	AFB smear result	Culture result	Reference LDT result	GeneXpert MTB/Rif result
Lymph node	Negative	Growth of MTB	Not done	Negative
Lymph node, neck	Negative	Growth of MTB	Not done	Negative
Endometrial biopsy	Negative	Growth of MTB	Not done	Negative
Lung wedge	No concurrent fresh sample, AFB seen on histopathology	Not done	MTB detected	Negative
L4/5 paraspinal biopsy+aspirate	Negative	Growth of MTB	Not done	Negative
T11 bone biopsy	Few AFB	Growth of MTB	Not done	Negative
Lymph node, right supraclavicular	No concurrent fresh sample, no AFB on histopathology	Not done	MTB detected	Negative
EBUS, lymph node	No concurrent fresh sample, no AFB on histopathology	Growth of MTB	MTB detected	Negative
Lung biopsy	Negative	Growth of MTB	Indeterminate	Negative

EBUSEndobrochial Ultrasound

For the remaining 19 negative specimens, 5 were confirmed MTB negative by culture. Two of those five culture-negative specimens had tissue sections positive for AFB; however, culture grew NTM: *M. malmoense* in lung tissue and *M. haemophilum* in a skin biopsy (species identification was confirmed via targeted sequencing of 401 bp fragment of *hsp65* gene at our provincial reference laboratory [11]). Of the 19 negative specimens, 16 were confirmed negative for MTB by reference laboratory LDT MTB PCR and 3 had no LDT MTB PCR results available; some specimens overlapped, with some having both negative MTB culture results and negative LDT MTB PCR results. All 19 specimens tested negative by the Xpert MTB/Rif Ultra assay, with a clinical specificity of 100% (95% CI of 83.2–100.0%). Details are given in Table 3.

**Table 3.** Sample distribution type in correlation with negative culture and/or LDT result (*n*=19)

Sample type	Total count	AFB positive	MTB culture negative	LDT MTB PCR negative	GeneXpert MTB/Rif Ultra assay, with specificity (SPC) and 95% CI
Lymph nodes	5	1	1	4	No MTB in 19 of 19 negative specimens (SPC=100%, 83.2–100.0%)
Lung (parenchymal or pleural tissue)	8	0*	3	7	
Musculoskeletal/joint	3	n/a	0	3	
Dermatologic	3	1†	1	2	

* Of the 8eight lung tissue samples, only 1one had smear result available on fresh tissue. It was AFB negative, and culture grew *M. malmoense* (identified via targeted sequencing of 401 bp fragment of the *hsp65* gene).

† Of the 3three skin/dermatologic samples, only 1one had smear result available on fresh tissue. It was AFB- positive, and culture grew *M. haemophilum* (identified via targeted sequencing of 401 bp fragment of the *hsp65* gene).

Chi-square test of independence was performed to assess the relationship between mycobacterial culture and our Xpert MTB/Rif Ultra results. There was a significant relationship between the two variables, *X*^2^(1, 26)=7.22, *P*=7.20×10^−3^. Likewise, when assessing the reference laboratory LDT MTB PCR against our Xpert MTB/Rif Ultra results, there was also a significant relationship between both variables, *X*^2^ (1, 27)=19.64, *P*=9.37×10^−6^.

## Discussion

Despite advances in the diagnosis of pulmonary TB, rapid and reliable diagnosis of EPTB remains challenging due to the paucibacillary nature of infection and often lack of fresh tissue samples for culture. The current study evaluated the performance of the Xpert MTB/Rif Ultra assay for the detection of MTB in FFPET specimens. The Xpert MTB/Rif Ultra assay demonstrated 71.9% sensitivity (95% CI 54.6–84.4%) relative to culture and/or reference laboratory MTB LDT assay, and none of the specimens tested positive for rifampin resistance, which was concordant for all specimens with available phenotypic susceptibility results. Of the nine false-negative specimens on Xpert MTB/Rif Ultra assay, one had ‘few’ AFBs reported on fresh specimen smear and another had AFB seen on histopathological examination of the fixed tissue. The remaining seven were negative for AFB.

Upon review of previous studies evaluating the use of MTB PCR on FFPET, reported test sensitivities have been variable. For instance, a Kenyan study using histology as the reference standard had MTB PCR sensitivity of 53.2% (95% CI 41.6–64.9%) on archived FFPET with histological features consistent with TB. However, the researchers were not able to comment on specificity, as no concurrent tissue culture results were available [5]. In terms of evaluation of FFPET via molecular methods, many studies utilize CRS integrating clinical features and response to anti-tuberculous therapy, cytology and radiographic findings, given the limitations with culture dependent on sample volume and quality along with paucibacillary nature of EPTB [12,14]. Chaudhary *et al*. acknowledged that when Xpert Ultra was compared with CRS, highly variable sensitivities were noted (ranging 18–34% in urine, 37–61% in pleural fluid, 27–70% in lymph node tissue/aspirate and 33–95% in CSF); specificities ranged 95–99% depending on whether culture or CRS was used as a reference standard [14]. Gu *et al*. noted that histopathology results alone had 92.9% sensitivity based on liquid culture test of biopsy samples (compared with 83.3% for Xpert MTB/Rif), but had noticeably lower specificity (45.21% versus 63.01% for Xpert, respectively) and suggested a combination of histopathology with molecular results to improve diagnostic evaluation of EPTB samples [13]. Another study evaluating 61 FFPET had a sensitivity of 85.7% (36/42 positive samples) and 100% specificity with repeat extraction showing improvement to 39 concordant positives (i.e. 92.9%); however, it compared the previous Xpert MTB/Rif assay with a reference laboratory LDT PCR assay targeting *IS6110* and did not include MTB culture as a reference method [9]. Finally, a study in China did a parallel comparison of the Xpert MTB/Rif assay with Xpert MTB/Rif Ultra on 83 FFPET tissues (MTB confirmed either on culture or previous MTB PCR); Xpert MTB/Rif Ultra consistently showed significantly higher sensitivity of 97.6% (95% CI 92.6–99.5%) regardless of pulmonary or extra-pulmonary sources [15]. A 2023 review article by Dahiya *et al*. supports these findings, where they noted increased sensitivity, particularly in cases of TB lymphadenitis, pleuritis and meningitis, with the caveat remaining that there is a limited sample size to fully evaluate other clinical EPTB manifestations [16]. Of note, Xpert MTB/Rif Ultra reportedly has a limit of detection of 16 c.f.u. of MTB ml^−1^ compared to 114 c.f.u. ml^−1^ for Xpert MTB/Rif [17]. This improved LOD (limit of detection) could be related to the updated assay design [addition of multicopy number *IS6110* and *IS1081* targets to the *rpoB* target and nested (Xpert MTB/Rif Ultra) versus semi-nested (Xpert MTB/Rif) PCR]. Although the performance characteristics were determined, using spiked sputum samples might not be directly transferable to clinical EPTB samples. Nonetheless, the updated assay design might also explain the improved performance reported for the Ultra assay in EPTB.

Formalin fixation and paraffin embedding preserve tissue morphology but also result in nucleic acid fragmentation and therefore can lead to lower detection sensitivity. Sampling variability, exhaustion of tissue from the paraffin block, DNA degradation during storage and extraction variability may explain the false-negative results observed in the current study. In addition, the Xpert MTB/Rif Ultra cartridge is used to extract DNA from intact bacteria in fresh specimens and any free DNA from the processing of tissue sections may be lost during extraction. The assay specificity was 100% when compared with MTB culture or reference laboratory MTB LDT assay.

The current study strengths include the diverse FFPET specimen types, which support the universal application of this method across EPTB sites irrespective of the organ system affected. Furthermore, the assay is relatively easy to implement in clinical laboratories given the simple processing procedure and minimal hands-on time. Furthermore, FFPET processing inactivates any viable mycobacteria, which eliminates the requirement for containment level 3 facilities and allows non-reference laboratories to perform the assay. Compared to external reference testing, MTB PCR results by Xpert MTB/Rif Ultra are readily available within 24 h along with information regarding genotypic rifampin resistance, which may facilitate earlier intervention for infection prevention and control purposes and modification of anti-tuberculous treatment.

Limitations included a small sample size, as not all the FFPET had enough residual specimens to do Xpert MTB/Rif Ultra testing and a lack of concurrent fresh tissue for MTB culture for LDT MTB PCR-negative specimens. Given the paucibacillary nature of EPTB, tissue sections can have significant variability in microbial load, and some might not have adequate organism present – utilizing sections that might not have had an adequate amount of microorganisms present may explain the false-negative samples in the current study. It is important to note that Xpert does not have an endogenous control target for testing formalin-fixed tissue scrolls, and hence, it is very difficult to determine if the selected tissue scrolls actually contained any tissue or human cells.

Finally, although not all cases had fresh samples collected for MTB culture and susceptibility testing, we correlated all specimens with available results for phenotypic drug susceptibilities. There were no rifampin resistance discrepancies between genotypic and phenotypic drug susceptibilities regardless of smear positivity (all Xpert MTB/Rif Ultra results were negative for rifampin resistance).

The current study helps extend and support the implementation of molecular testing in facilitating and expediting the diagnosis of EPTB disease. Additional process improvements between anatomical pathology and microbiology laboratories would include determining optimum FFPET scroll thickness, particularly when reviewing formalin-fixed tissues with histologic findings suggestive of MTB disease. Optimizing the communication processes between histopathology and microbiology teams (e.g. joint rounds to review specimen section blocks) could also help with optimizing tissue yield for successful molecular microbiologic testing.

It is prudent for clinicians to review the need for mycobacterial culture based on the risk factors at the time of invasive specimen collection to prevent scenarios where formalin-fixed tissues are the sole specimen type available. Without cultured fresh tissue specimens, there are essentially no established methods at present to do proper susceptibility testing for mycobacteria nor genotypic epidemiology surveillance [18], ultimately resulting in decreased quality of care and delayed treatment for patients with EPTB disease.

## Abbreviations

AFB: acid-fast bacilli
BCCDC: British Columbia Centre for Disease Control
CI: confidence interval
CL3: containment level 3
CRS: composite reference standard
EBUS: endobronchial ultrasound
EPTB: extra-pulmonary tuberculosis
FDA: food and drug administration
FFPET: formalin-fixed paraffin-embedded tissue
LDT: laboratory-developed test
LOD: limit of detection
NTM: non-tuberculous mycobacteria
SPC: specificity
TB: tuberculosis
